# Supplementary material for: Effects of wine-cap Stropharia cultivation on soil nutrients and bacterial communities in forestlands of northern China
Source: PeerJ. 2018 Oct 9;6:e5741. doi: 10.7717/peerj.5741 (PMC6183509; doi:10.7717/peerj.5741)
Supplement: Table S1 [file peerj-06-5741-s001.docx]

**Table S1.** Alpha diversity index (α-diversity index) for the bacterial community among different grids.

| α-Diversity index | Sample plot groups | | | | |
| --- | --- | --- | --- | --- | --- |
|  | Y000 | Y010 | Y011 | Y001 | Y101 |
| OTUs^1^ | 5011±105a | 4859±57a | 4948±92a | 5005±265a | 4756±117a |
| OTUs^2^ | 4379±82a | 4246±62a | 4312±75a | 4350±176a | 4109±90a |
| Coverage^3^ | 0.9847±0.003a | 0.9843±0.003a | 0.985±0.006a | 0.9857±0.027a | 0.9847±0.003a |
| Shannon^4^ | 10.21±0.084a | 9.98±0.09a | 10.1±0.004a | 10.02±0.088a | 9.95±0.098a |
| Simpson^5^ | 0.9973±0.0007a | 0.9967±0.0003a | 0.997a | 0.9967±0.0003a | 0.9963±0.0003a |

Values are the mean ± standard deviation (N=3). Values within the same row followed by the same letter are not significantly different at *P*>0.05 (ANOVA, Tukey’s analysis). ^1^ OTUs: number of operational taxonomic units (97% similarity) before normalization. ^2^ OTUs: number of operational taxonomic units (97% similarity) after normalization. ^3^ Coverage: Good’s coverage estimator. ^4^ Shannon: Shannon index. ^5^ Simpson: Simpson index.
