# Supplementary material for: Effects of wine-cap Stropharia cultivation on soil nutrients and bacterial communities in forestlands of northern China
Source: PeerJ. 2018 Oct 9;6:e5741. doi: 10.7717/peerj.5741 (PMC6183509; doi:10.7717/peerj.5741)
Supplement: Table S2 [file peerj-06-5741-s002.docx]

**Table S2.** Relative abundance of the top ten dominant phyla among different grids.

| Phyla | Y000 | Y010 | Y011 | Y001 | Y101 |
| --- | --- | --- | --- | --- | --- |
| Proteobacteria | 38.63±2.38a | 38.22±1.49a | 36.29±1.08a | 36.97±0.85a | 41.38±1.66a |
| **Acidobacteria** | **18.49±0.33b** | **22.25±0.91ab** | **21.22±0.46ab** | **22.63±1.46a** | **20.44±0.54ab** |
| Actinobacteria | 9.42±0.89a | 7.08±0.45a | 9.28±1.48a | 8.23±0.68a | 6.72±0.31a |
| Chloroflexi | 7.73±0.86a | 6.35±0.62a | 6.35±0.48a | 6.60±0.23a | 5.34±0.66a |
| **Planctomycetes** | **4.84±0.29b** | **5.47±0.11b** | **5.32±0.18b** | **5.83±0.14ab** | **6.73±0.44a** |
| Bacteroidetes | 3.58±0.74a | 5.03±0.78a | 4.15±0.71a | 3.94±0.25a | 6.29±0.65a |
| **Gemmatimonadetes** | **4.24±0.1a** | **4.83±0.72a** | **4.91±0.28a** | **4.96±0.14a** | **2.94±0.25b** |
| Firmicutes | 2.84±0.63a | 1.76±0.06a | 2.39±0.33a | 2.00±0.18a | 2.52±0.52a |
| Nitrospirae | 3.39±0.22a | 2.69±0.34a | 3.04±0.18a | 2.80±0.52a | 2.19±0.21a |
| Chlorobi | 0.55±0.17a | 0.59±0.13a | 1.24±0.32a | 0.42±0.12a | 0.40±0.13a |

Values are mean ± standard deviation (N = 3). Values within the same row followed by the same letter are not significantly different at P > 0.05 (ANOVA, Tukey analysis). The phyla with significant change in relative abundance are shown in bold.
