# Supplementary material for: Effects of wine-cap Stropharia cultivation on soil nutrients and bacterial communities in forestlands of northern China
Source: PeerJ. 2018 Oct 9;6:e5741. doi: 10.7717/peerj.5741 (PMC6183509; doi:10.7717/peerj.5741)
Supplement: Table S3 [file peerj-06-5741-s003.docx]

**Table S3.** Number of significantly different bacterial community groups by pairwise comparison between forestlands cultivated with *Stropharia rugosoannulata* (Y010, Y011, Y001 and Y101) and the no-cultivation control (Y000).

| Comparison pairs | Y010 | Y011 | Y001 | | Y101 | Y000 |
| --- | --- | --- | --- | --- | --- | --- |
| Y010/Y000 | 3 |  | |  |  | 0 |
| Y011/Y000 |  | 2 |  | |  | 0 |
| Y001/Y000 |  |  | 3 | |  | 1 |
| Y101/Y000 |  |  |  | | 4 | 1 |
