# Supplementary material for: Effects of wine-cap Stropharia cultivation on soil nutrients and bacterial communities in forestlands of northern China
Source: PeerJ. 2018 Oct 9;6:e5741. doi: 10.7717/peerj.5741 (PMC6183509; doi:10.7717/peerj.5741)
Supplement: Table S4 [file peerj-06-5741-s004.docx]

**Table S4.** The tests of AMOVA (analysis of molecular variance), ANOSIM (analysis of similarities) and ADONIS (PERMANOVA, permutational multivariate analysis of variance) for comparing bacterial community in each grid.

| Comparison pairs | AMOVA | | ANOSIM | | ADONIS | |
| --- | --- | --- | --- | --- | --- | --- |
|  | ^1^ Fs | ^2^ *P* | ^3^ R | *P* | ^4^ R2 | *P* |
| Y000-Y010 | 2.26253 | 0.056 | 1 | 0.1 | 0.3949 | 0.001389 |
| Y000-Y011 | 1.57134 | 0.22 | 0.9259 | 0.1 | 0.35335 | 0.001389 |
| Y000-Y001 | 2.8878 | 0.041 | 1 | 0.1 | 0.43731 | 0.001389 |
| Y000-Y101 | 4.05419 | 0.074 | 1 | 0.1 | 0.52097 | 0.1 |
| Y010-Y011 | 1.0906 | 0.298 | 0.1481 | 0.2 | 0.25063 | 0.1014 |
| Y010-Y001 | 1.2292 | 0.522 | 0.6296 | 0.1 | 0.30909 | 0.001389 |
| Y010-Y101 | 2.50983 | 0.102 | 1 | 0.1 | 0.48237 | 0.001389 |
| Y011-Y001 | 1.96764 | 0.199 | 0.8519 | 0.1 | 0.37537 | 0.001389 |
| Y011-Y101 | 3.26033 | 0.099 | 1 | 0.1 | 0.52237 | 0.1 |
| Y001-Y101 | 3.27186 | 0.097 | 1 | 0.1 | 0.46865 | 0.001389 |

^1^ Fs: F test value. ^2^ *P*: p-value, the p-value<0.05 indicate the significant difference of bacterial community in each comparison pairs. ^3^ R: R-value is between (-1,1), R-value>0 indicate that there are significant differences between groups, R-value<0 indicate that the difference within the grid is greater than the difference between the grids. ^4^ R2: R2-value, which indicates the degree of interpretation of sample differences by different grids. The larger R2-value is, the higher the degree of interpretation of the difference by the grids.
