# Supplementary figures and images for: Effects of wine-cap Stropharia cultivation on soil nutrients and bacterial communities in forestlands of northern China
Source: PeerJ. 2018 Oct 9;6:e5741. doi: 10.7717/peerj.5741 (PMC6183509; doi:10.7717/peerj.5741)

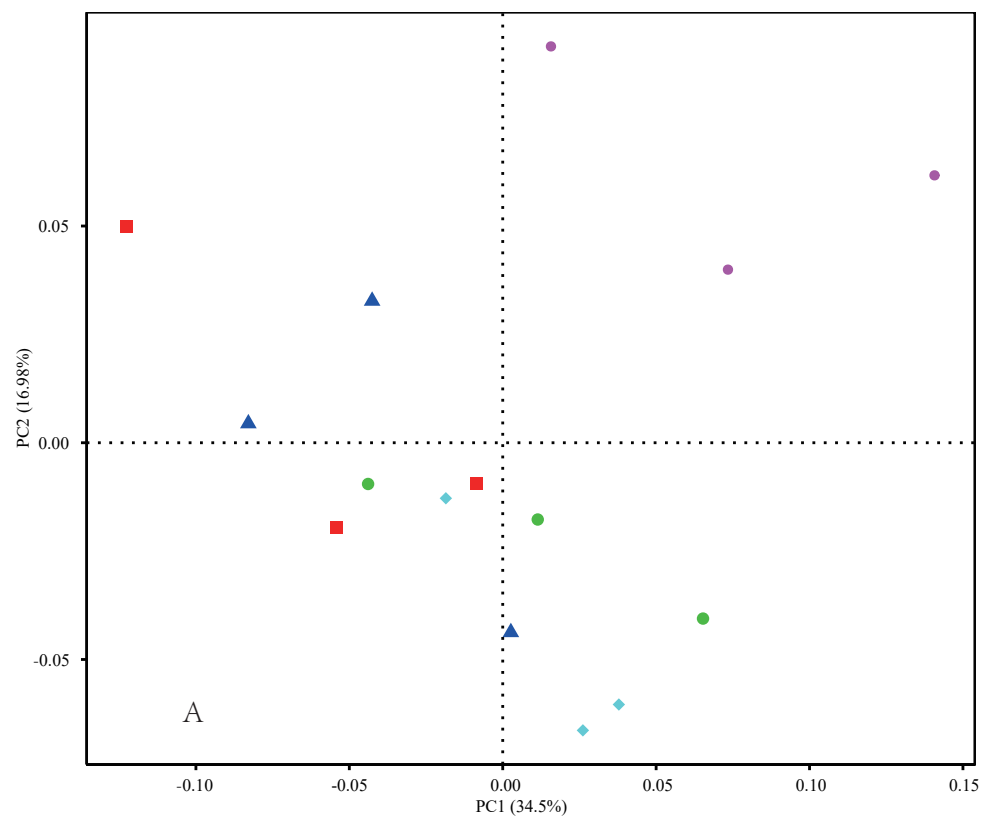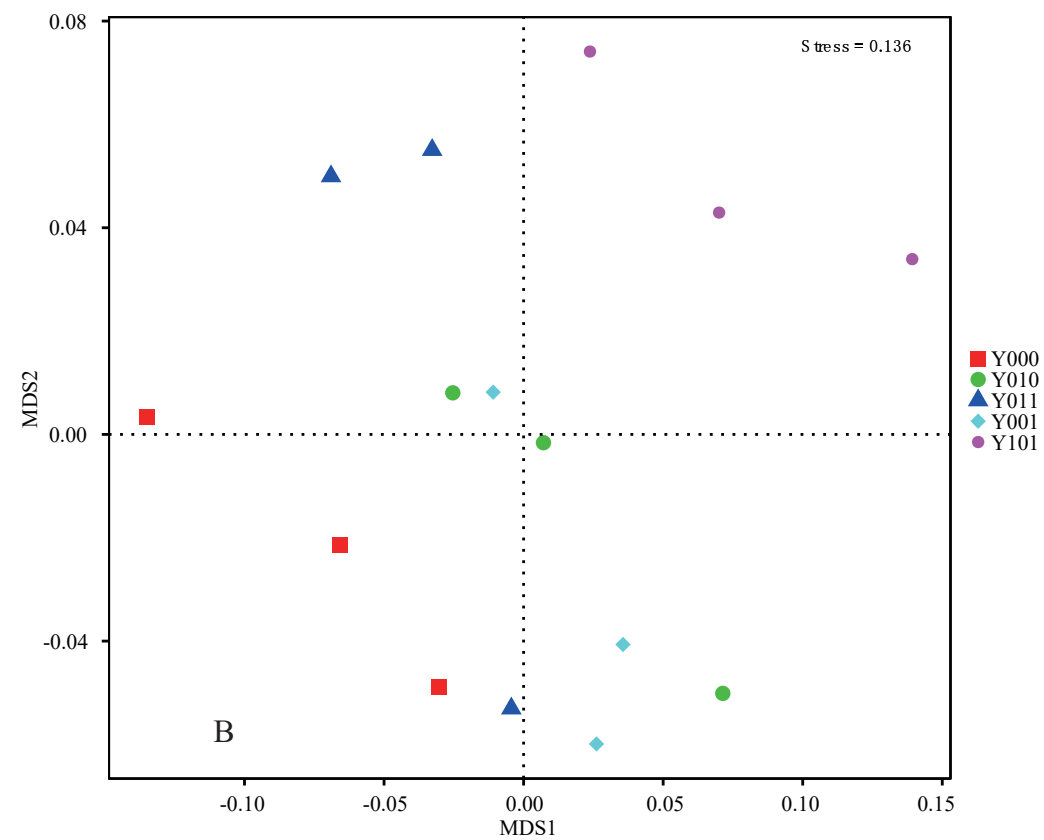

Supplement: Figure S1 — (A) Principal Co-ordinates Analysis (PCoA). (B) Non-Metric Multi-Dimensional Scaling (NMDS). Different grids are represented by different colors. Spots with the same color represent the same grids. [file peerj-06-5741-s005.pdf]

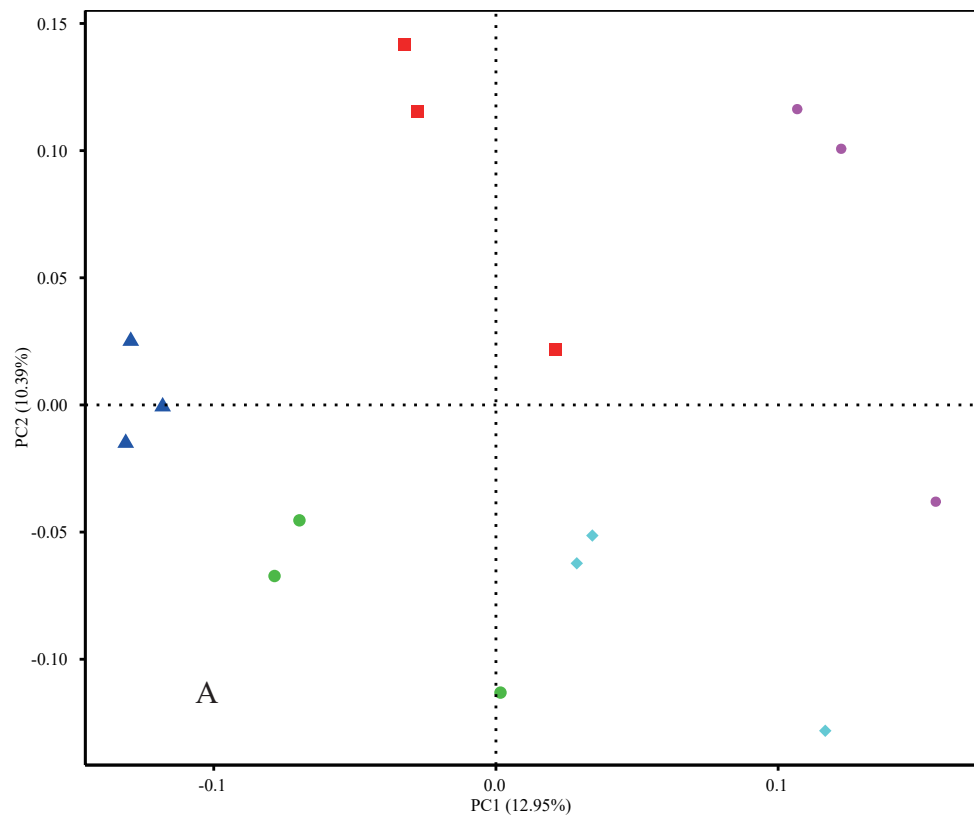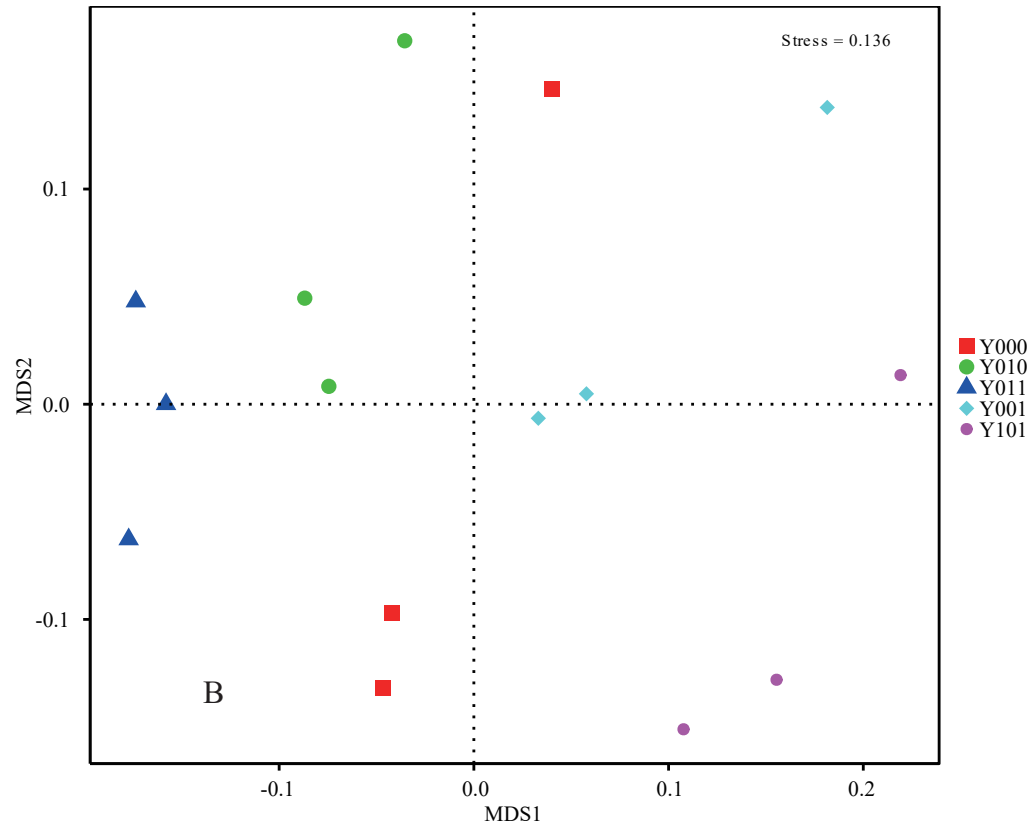

Supplement: Figure S2 — (A) Principal Co-ordinates Analysis (PCoA). (B) Non-Metric Multi-Dimensional Scaling. Different grids are represented by different colors. Spots with the same color represent the same grids. [file peerj-06-5741-s006.pdf]

eria  
b--Nitrospirae

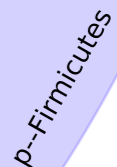

p--Bacteroidetes

Supplement: Figure S6 — The color of the branch represents its corresponding phylum, and each color represents a phylum. The size of the circle is proportional to the abundance of the taxonomic groups. The top 40 taxonomic groups in abundance are represented by solid circles. [file peerj-06-5741-s010.pdf]
