# Supplementary material for: Effects of wine-cap Stropharia cultivation on soil nutrients and bacterial communities in forestlands of northern China
Source: PeerJ. 2018 Oct 9;6:e5741. doi: 10.7717/peerj.5741 (PMC6183509; doi:10.7717/peerj.5741)
Supplement: Figure S3 — The color of the branch represents its corresponding phylum, and each color represents a phylum. The size of the circle is proportional to the abundance of the taxonomic groups. The top 40 taxonomic groups in abundance are represented by solid circles. [file peerj-06-5741-s007.pdf]

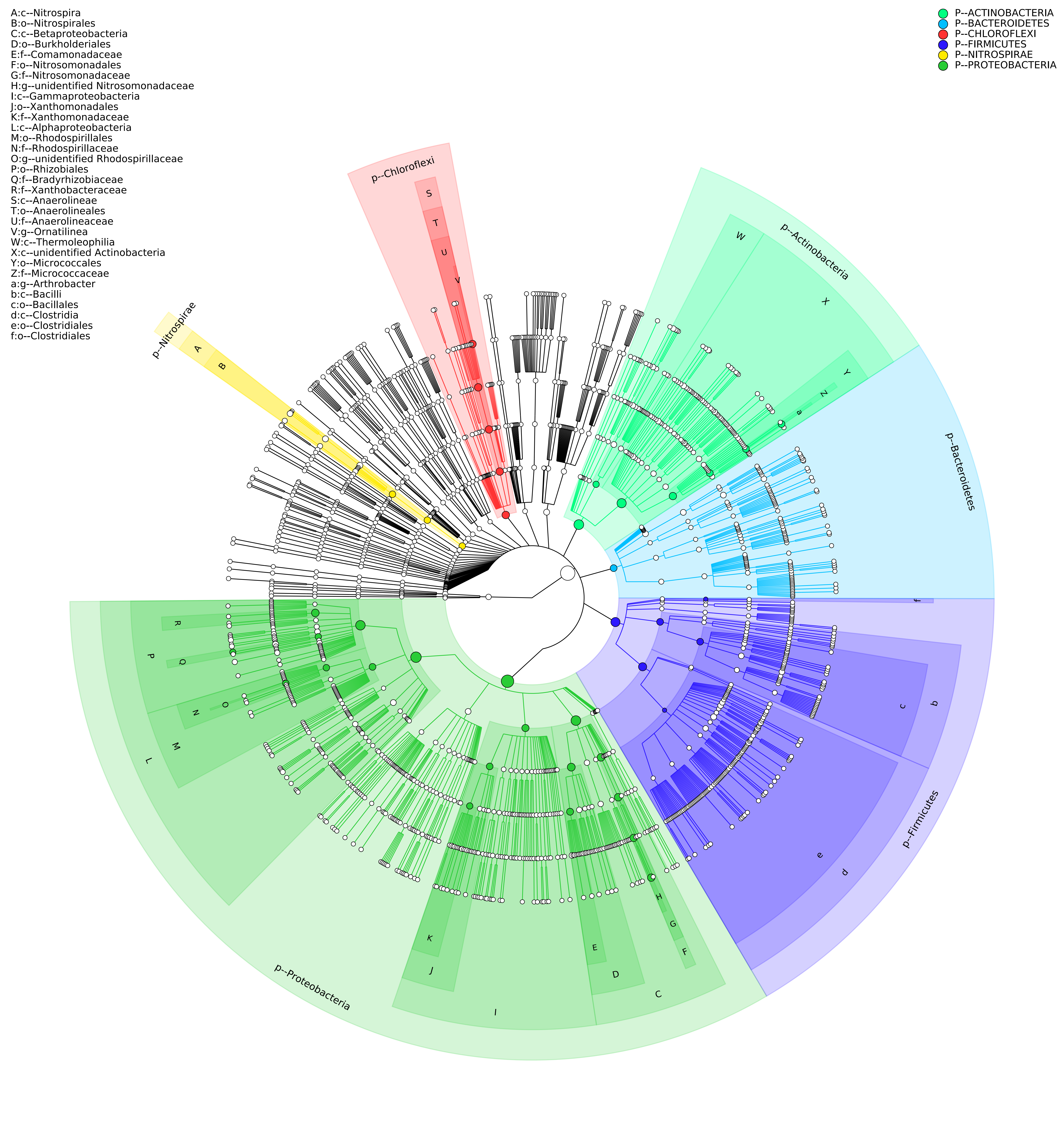
A:c--Nitrospira
B:o--Nitrospirales
C:c--Betaproteobacteria
D:o--Burkholderiales
E:f--Comamonadaceae
F:o--Nitrosomonadales
G:f--Nitrosomonadaceae
H:g--unidentified Nitrosomonadaceae
I:c--Gammaproteobacteria
J:o--Xanthomonadales
K:f--Xanthomonadaceae
L:c--Alphaproteobacteria
M:o--Rhodospirillales
N:f--Rhodospirillaceae
O:g--unidentified Rhodospirillaceae
P:o--Rhizobiales
Q:f--Bradyrhizobiaceae
R:f--Xanthobacteraceae
S:c--Anaerolineae
T:o--Anaerolineales
U:f--Anaerolineaceae
V:g--Ornatilinea
W:c--Thermoleophilia
X:c--unidentified Actinobacteria
Y:o--Micrococcales
Z:f--Micrococcaceae
a:g--Arthrobacter
b:c--Bacilli
c:o--Bacillales
d:c--Clostridia
e:o--Clostridiales
f:o--Clostridiales
P--ACTINOBACTERIA
P--BACTEROIDETES
P--CHLOROFLEXI
P--FIRMICUTES
P--NITROSPIRAE
P--PROTEOBACTERIA
p--Chloroflexi
p--Nitrospirae
p--Actinobacteria
p--Bacteroidetes
p--Firmicutes
p--Proteobacteria
