# Supplementary material for: Effects of wine-cap Stropharia cultivation on soil nutrients and bacterial communities in forestlands of northern China
Source: PeerJ. 2018 Oct 9;6:e5741. doi: 10.7717/peerj.5741 (PMC6183509; doi:10.7717/peerj.5741)
Supplement: Figure S4 — The color of the branch represents its corresponding phylum, and each color represents a phylum. The size of the circle is proportional to the abundance of the taxonomic groups. The top 40 taxonomic groups in abundance are represented by solid circles. [file peerj-06-5741-s008.pdf]

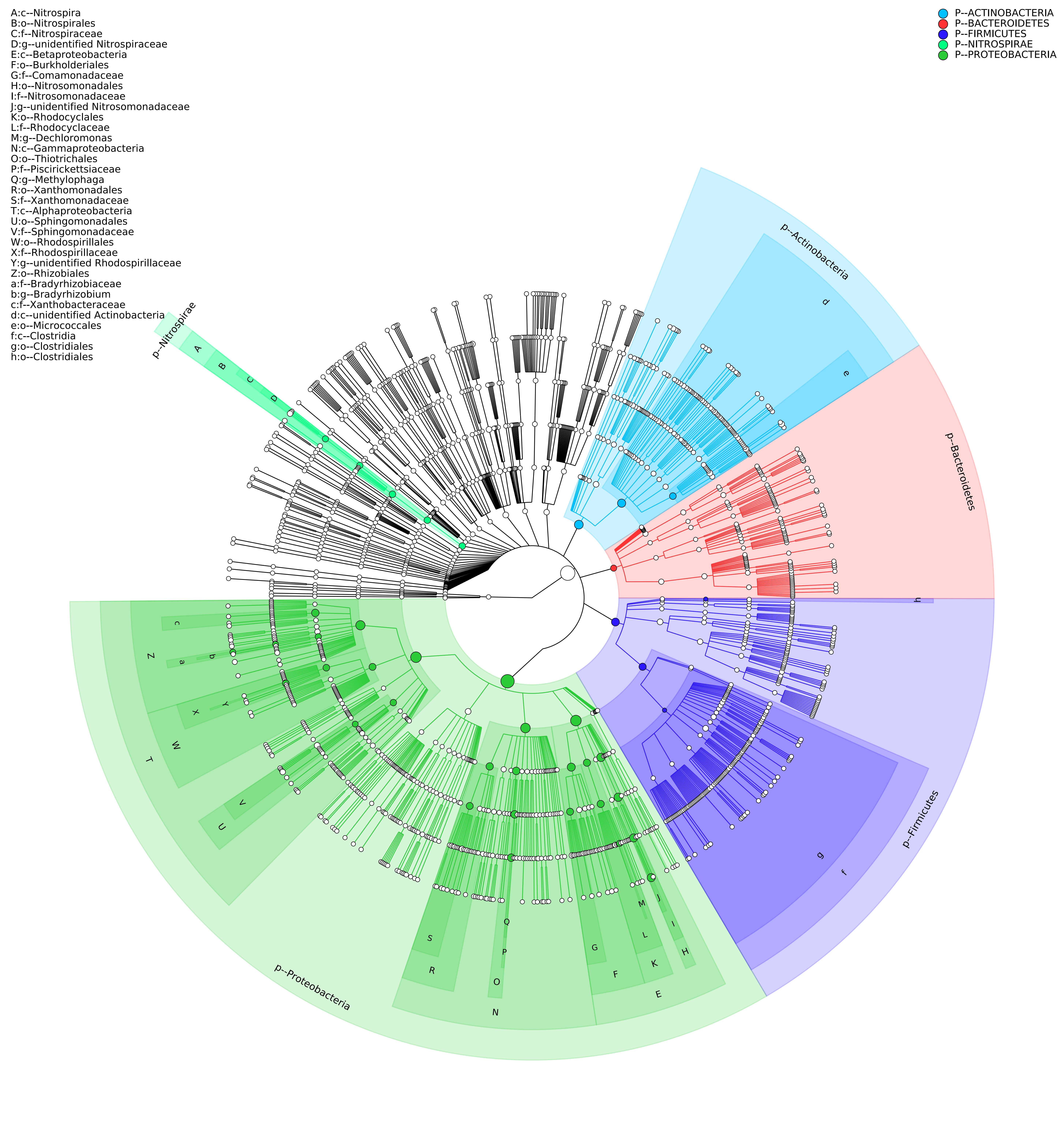
A:c--Nitrospira
B:o--Nitrospirales
C:f--Nitrospiraceae
D:g--unidentified Nitrospiraceae
E:c--Betaproteobacteria
F:o--Burkholderiales
G:f--Comamonadaceae
H:o--Nitrosomonadales
I:f--Nitrosomonadaceae
J:g--unidentified Nitrosomonadaceae
K:o--Rhodocyclales
L:f--Rhodocyclaceae
M:g--Dechloromonas
N:c--Gammaproteobacteria
O:o--Thiotrichales
P:f--Piscirickettsiaceae
Q:g--Methylophaga
R:o--Xanthomonadales
S:f--Xanthomonadaceae
T:c--Alphaproteobacteria
U:o--Sphingomonadales
V:f--Sphingomonadaceae
W:o--Rhodospirillales
X:f--Rhodospirillaceae
Y:g--unidentified Rhodospirillaceae
Z:o--Rhizobiales
a:f--Bradyrhizobiaceae
b:g--Bradyrhizobium
c:f--Xanthobacteraceae
d:c--unidentified Actinobacteria
e:o--Micrococcales
f:c--Clostridia
g:o--Clostridiales
h:o--Clostridiales
P--ACTINOBACTERIA
P--BACTEROIDETES
P--FIRMICUTES
P--NITROSPIRAE
P--PROTEOBACTERIA
p--Nitrospirae
p--Actinobacteria
p--Bacteroidetes
p--Firmicutes
p--Proteobacteria
