# Supplementary material for: Effects of wine-cap Stropharia cultivation on soil nutrients and bacterial communities in forestlands of northern China
Source: PeerJ. 2018 Oct 9;6:e5741. doi: 10.7717/peerj.5741 (PMC6183509; doi:10.7717/peerj.5741)
Supplement: Figure S5 — The color of the branch represents its corresponding phylum, and each color represents a phylum. The size of the circle is proportional to the abundance of the taxonomic groups. The top 40 taxonomic groups in abundance are represented by solid circles. [file peerj-06-5741-s009.pdf]

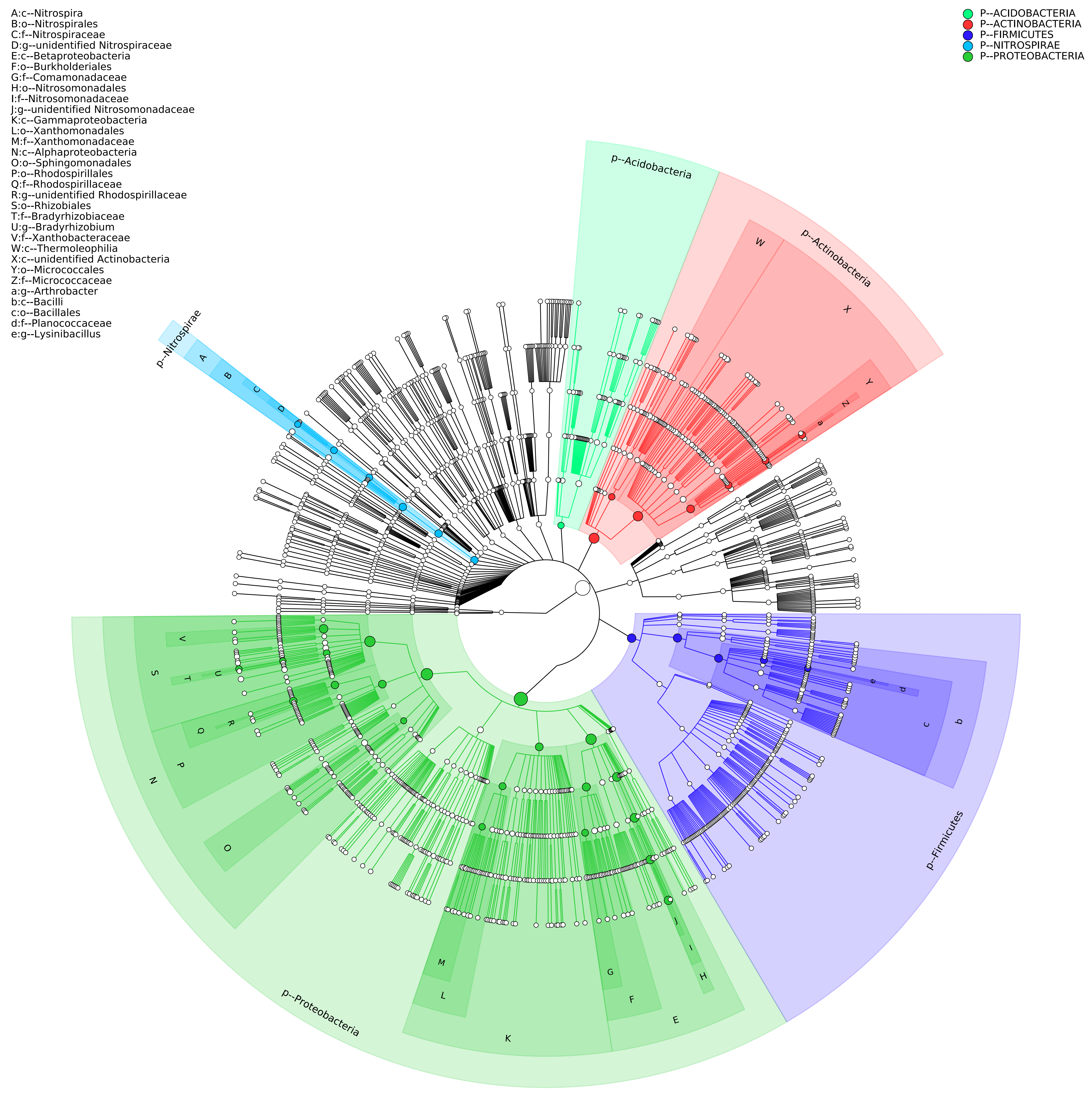
A:c--Nitrospira
B:o--Nitrospirales
C:f--Nitrospiraceae
D:g--unidentified Nitrospiraceae
E:c--Betaproteobacteria
F:o--Burkholderiales
G:f--Comamonadaceae
H:o--Nitrosomonadales
I:f--Nitrosomonadaceae
J:g--unidentified Nitrosomonadaceae
K:c--Gammaproteobacteria
L:o--Xanthomonadales
M:f--Xanthomonadaceae
N:c--Alphaproteobacteria
O:o--Sphingomonadales
P:o--Rhodospirillales
Q:f--Rhodospirillaceae
R:g--unidentified Rhodospirillaceae
S:o--Rhizobiales
T:f--Bradyrhizobiaceae
U:g--Bradyrhizobium
V:f--Xanthobacteraceae
W:c--Thermoleophilia
X:c--unidentified Actinobacteria
Y:o--Micrococcales
Z:f--Micrococcaceae
a:g--Arthrobacter
b:c--Bacilli
c:o--Bacillales
d:f--Planococcaceae
e:g--Lysinibacillus
P--ACIDOBACTERIA
P--ACTINOBACTERIA
P--FIRMICUTES
P--NITROSPIRAE
P--PROTEOBACTERIA
p--Acidobacteria
p--Actinobacteria
p--Nitrospirae
p--Firmicutes
p--Proteobacteria
