# Supplementary material for: Effects of wine-cap Stropharia cultivation on soil nutrients and bacterial communities in forestlands of northern China
Source: PeerJ. 2018 Oct 9;6:e5741. doi: 10.7717/peerj.5741 (PMC6183509; doi:10.7717/peerj.5741)
Supplement: Figure S7 — The color of the branch represents its corresponding phylum, and each color represents a phylum. The size of the circle is proportional to the abundance of the taxonomic groups. The top 40 taxonomic groups in abundance are represented by solid circles. [file peerj-06-5741-s011.pdf]

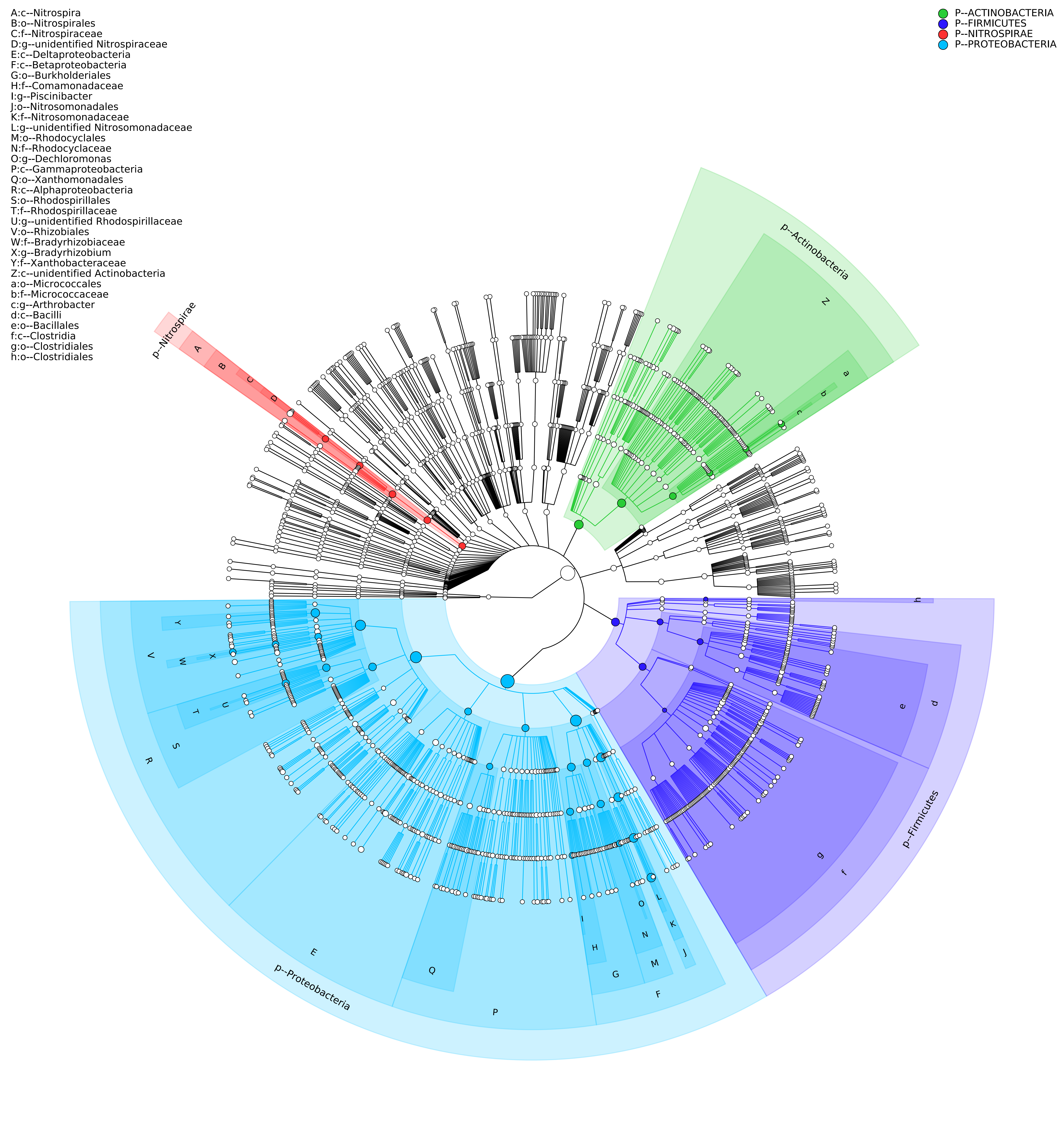
A:c--Nitrospira
B:o--Nitrospirales
C:f--Nitrospiraceae
D:g--unidentified Nitrospiraceae
E:c--Deltaproteobacteria
F:c--Betaproteobacteria
G:o--Burkholderiales
H:f--Comamonadaceae
I:g--Piscinibacter
J:o--Nitrosomonadales
K:f--Nitrosomonadaceae
L:g--unidentified Nitrosomonadaceae
M:o--Rhodocyclales
N:f--Rhodocyclaceae
O:g--Dechloromonas
P:c--Gammaproteobacteria
Q:o--Xanthomonadales
R:c--Alphaproteobacteria
S:o--Rhodospirillales
T:f--Rhodospirillaceae
U:g--unidentified Rhodospirillaceae
V:o--Rhizobiales
W:f--Bradyrhizobiaceae
X:g--Bradyrhizobium
Y:f--Xanthobacteraceae
Z:c--unidentified Actinobacteria
a:o--Micrococcales
b:f--Micrococcaceae
c:g--Arthrobacter
d:c--Bacilli
e:o--Bacillales
f:c--Clostridia
g:o--Clostridiales
h:o--Clostridiales
P--ACTINOBACTERIA
P--FIRMICUTES
P--NITROSPIRAE
P--PROTEOBACTERIA
p--Nitrospirae
p--Actinobacteria
p--Firmicutes
p--Proteobacteria
