# Supplementary material for: Effects of wine-cap Stropharia cultivation on soil nutrients and bacterial communities in forestlands of northern China
Source: PeerJ. 2018 Oct 9;6:e5741. doi: 10.7717/peerj.5741 (PMC6183509; doi:10.7717/peerj.5741)
Supplement: Figure S9 — The color of the branch represents its corresponding phylum, and each color represents a phylum. The size of the circle is proportional to the abundance of the taxonomic groups. The top 40 taxonomic groups in abundance are represented by solid circles. [file peerj-06-5741-s013.pdf]

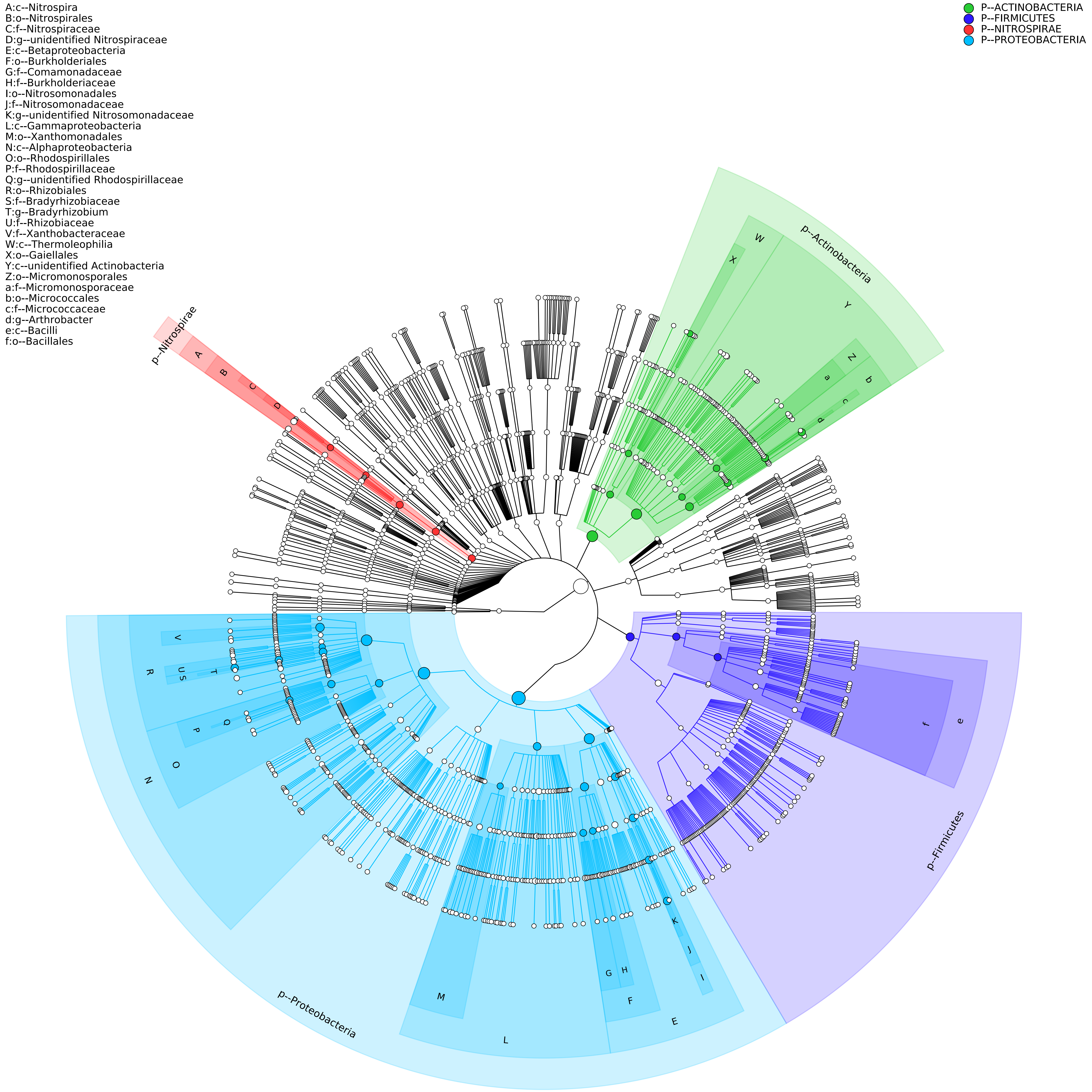
A:c--Nitrospira
B:o--Nitrospirales
C:f--Nitrospiraceae
D:g--unidentified Nitrospiraceae
E:c--Betaproteobacteria
F:o--Burkholderiales
G:f--Comamonadaceae
H:f--Burkholderiaceae
I:o--Nitrosomonadales
J:f--Nitrosomonadaceae
K:g--unidentified Nitrosomonadaceae
L:c--Gammaproteobacteria
M:o--Xanthomonadales
N:c--Alphaproteobacteria
O:o--Rhodospirillales
P:f--Rhodospirillaceae
Q:g--unidentified Rhodospirillaceae
R:o--Rhizobiales
S:f--Bradyrhizobiaceae
T:g--Bradyrhizobium
U:f--Rhizobiaceae
V:f--Xanthobacteraceae
W:c--Thermoleophilia
X:o--Gaiellales
Y:c--unidentified Actinobacteria
Z:o--Micromonosporales
a:f--Micromonosporaceae
b:o--Micrococcales
c:f--Micrococcaceae
d:g--Arthrobacter
e:c--Bacilli
f:o--Bacillales
P--ACTINOBACTERIA
P--FIRMICUTES
P--NITROSPIRAE
P--PROTEOBACTERIA
p--Nitrospirae
p--Actinobacteria
p--Firmicutes
p--Proteobacteria
