# Supplementary material for: Effects of wine-cap Stropharia cultivation on soil nutrients and bacterial communities in forestlands of northern China
Source: PeerJ. 2018 Oct 9;6:e5741. doi: 10.7717/peerj.5741 (PMC6183509; doi:10.7717/peerj.5741)
Supplement: Figure S10 — The color of the branch represents its corresponding phylum, and each color represents a phylum. The size of the circle is proportional to the abundance of the taxonomic groups. The top 40 taxonomic groups in abundance are represented by solid circles. [file peerj-06-5741-s014.pdf]

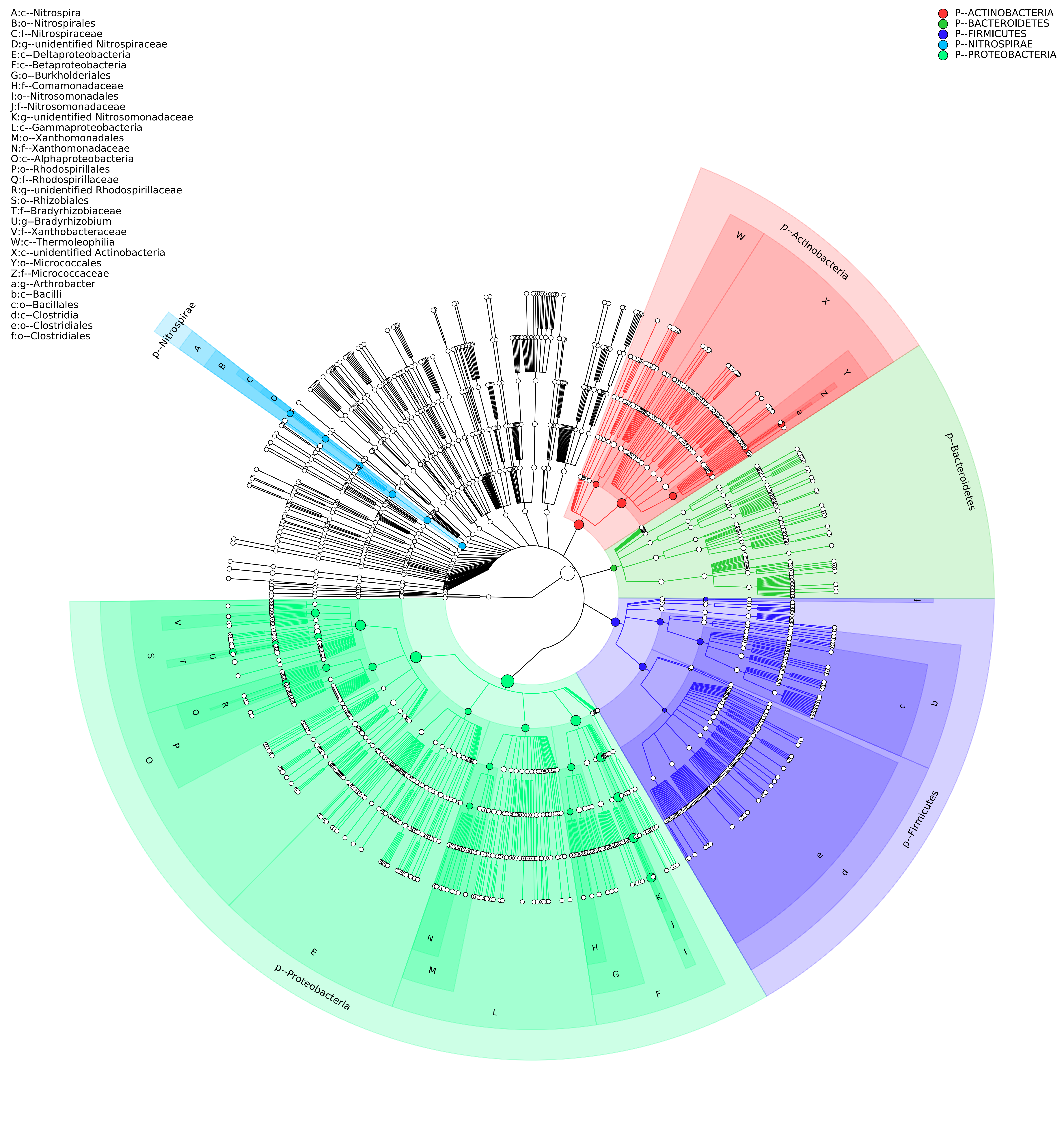
A:c--Nitrospira
B:o--Nitrospirales
C:f--Nitrospiraceae
D:g--unidentified Nitrospiraceae
E:c--Deltaproteobacteria
F:c--Betaproteobacteria
G:o--Burkholderiales
H:f--Comamonadaceae
I:o--Nitrosomonadales
J:f--Nitrosomonadaceae
K:g--unidentified Nitrosomonadaceae
L:c--Gammaproteobacteria
M:o--Xanthomonadales
N:f--Xanthomonadaceae
O:c--Alphaproteobacteria
P:o--Rhodospirillales
Q:f--Rhodospirillaceae
R:g--unidentified Rhodospirillaceae
S:o--Rhizobiales
T:f--Bradyrhizobiaceae
U:g--Bradyrhizobium
V:f--Xanthobacteraceae
W:c--Thermoleophilia
X:c--unidentified Actinobacteria
Y:o--Micrococcales
Z:f--Micrococcaceae
a:g--Arthrobacter
b:c--Bacilli
c:o--Bacillales
d:c--Clostridia
e:o--Clostridiales
f:o--Clostridiales
P--ACTINOBACTERIA
P--BACTEROIDETES
P--FIRMICUTES
P--NITROSPIRAE
P--PROTEOBACTERIA
p--Nitrospirae
p--Actinobacteria
p--Bacteroidetes
p--Firmicutes
p--Proteobacteria
