# Supplementary material for: Effects of wine-cap Stropharia cultivation on soil nutrients and bacterial communities in forestlands of northern China
Source: PeerJ. 2018 Oct 9;6:e5741. doi: 10.7717/peerj.5741 (PMC6183509; doi:10.7717/peerj.5741)
Supplement: Figure S11 — The color of the branch represents its corresponding phylum, and each color represents a phylum. The size of the circle is proportional to the abundance of the taxonomic groups. The top 40 taxonomic groups in abundance are represented by solid circles. [file peerj-06-5741-s015.pdf]

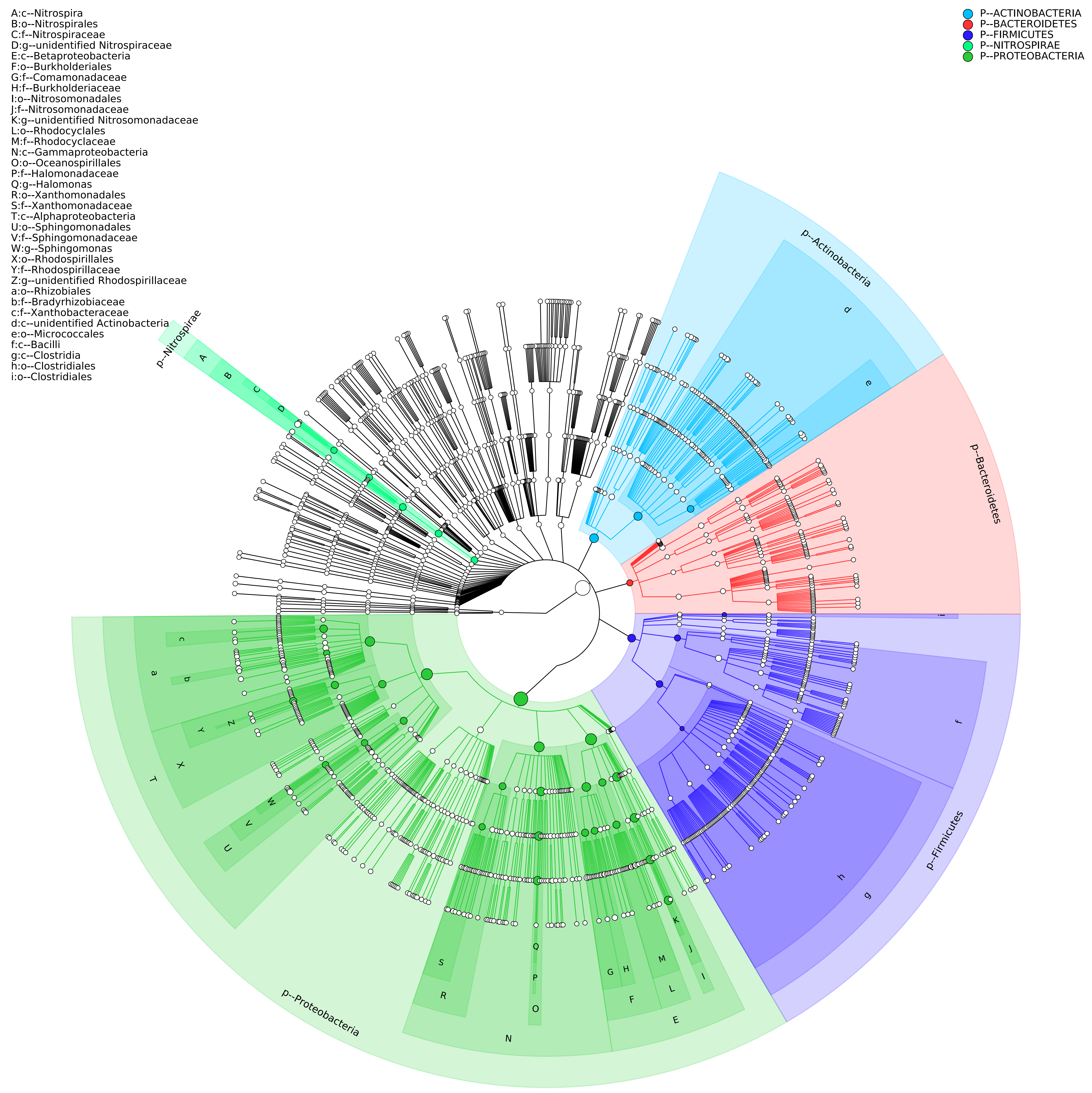
A:c--Nitrospira
B:o--Nitrospirales
C:f--Nitrospiraceae
D:g--unidentified Nitrospiraceae
E:c--Betaproteobacteria
F:o--Burkholderiales
G:f--Comamonadaceae
H:f--Burkholderiaceae
I:o--Nitrosomonadales
J:f--Nitrosomonadaceae
K:g--unidentified Nitrosomonadaceae
L:o--Rhodocyclales
M:f--Rhodocyclaceae
N:c--Gammaproteobacteria
O:o--Oceanospirillales
P:f--Halomonadaceae
Q:g--Halomonas
R:o--Xanthomonadales
S:f--Xanthomonadaceae
T:c--Alphaproteobacteria
U:o--Sphingomonadales
V:f--Sphingomonadaceae
W:g--Sphingomonas
X:o--Rhodospirillales
Y:f--Rhodospirillaceae
Z:g--unidentified Rhodospirillaceae
a:o--Rhizobiales
b:f--Bradyrhizobiaceae
c:f--Xanthobacteraceae
d:c--unidentified Actinobacteria
e:o--Micrococcales
f:c--Bacilli
g:c--Clostridia
h:o--Clostridiales
i:o--Clostridiales
P--ACTINOBACTERIA
P--BACTEROIDETES
P--FIRMICUTES
P--NITROSPIRAE
P--PROTEOBACTERIA
p--Nitrospirae
p--Actinobacteria
p--Bacteroidetes
p--Firmicutes
p--Proteobacteria
