# Supplementary material for: Effects of wine-cap Stropharia cultivation on soil nutrients and bacterial communities in forestlands of northern China
Source: PeerJ. 2018 Oct 9;6:e5741. doi: 10.7717/peerj.5741 (PMC6183509; doi:10.7717/peerj.5741)
Supplement: Figure S12 — The color of the branch represents its corresponding phylum, and each color represents a phylum. The size of the circle is proportional to the abundance of the taxonomic groups. The top 40 taxonomic groups in abundance are represented by solid circles. [file peerj-06-5741-s016.pdf]

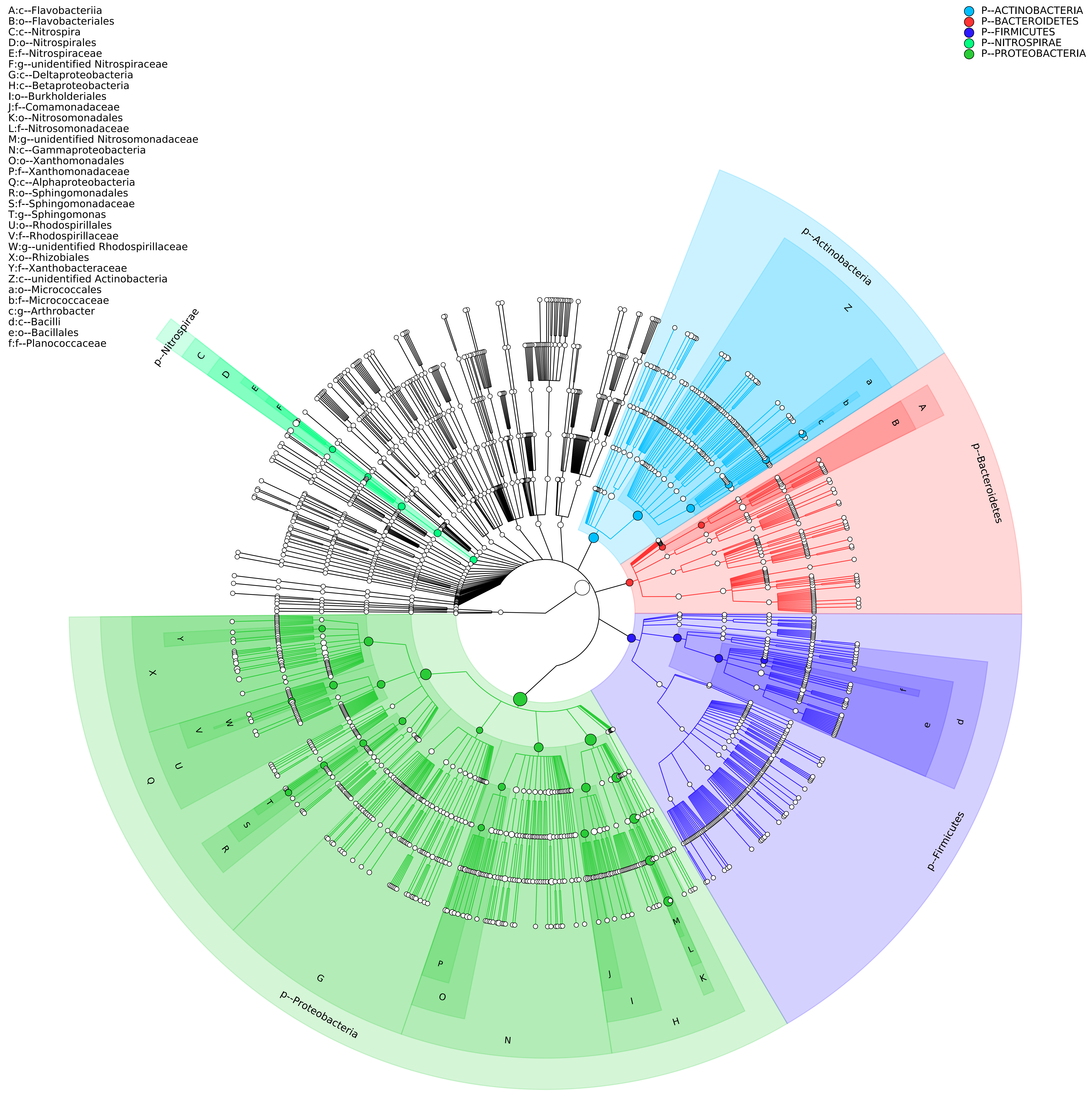
A:c--Flavobacteriia
B:o--Flavobacteriales
C:c--Nitrospira
D:o--Nitrospirales
E:f--Nitrospiraceae
F:g--unidentified Nitrospiraceae
G:c--Deltaproteobacteria
H:c--Betaproteobacteria
I:o--Burkholderiales
J:f--Comamonadaceae
K:o--Nitrosomonadales
L:f--Nitrosomonadaceae
M:g--unidentified Nitrosomonadaceae
N:c--Gammaproteobacteria
O:o--Xanthomonadales
P:f--Xanthomonadaceae
Q:c--Alphaproteobacteria
R:o--Sphingomonadales
S:f--Sphingomonadaceae
T:g--Sphingomonas
U:o--Rhodospirillales
V:f--Rhodospirillaceae
W:g--unidentified Rhodospirillaceae
X:o--Rhizobiales
Y:f--Xanthobacteraceae
Z:c--unidentified Actinobacteria
a:o--Micrococcales
b:f--Micrococcaceae
c:g--Arthrobacter
d:c--Bacilli
e:o--Bacillales
f:f--Planococcaceae
P--ACTINOBACTERIA
P--BACTEROIDETES
P--FIRMICUTES
P--NITROSPIRAE
P--PROTEOBACTERIA
p--Actinobacteria
p--Bacteroidetes
p--Firmicutes
p--Proteobacteria
p--Nitrospirae
