# Supplementary material for: Effects of wine-cap Stropharia cultivation on soil nutrients and bacterial communities in forestlands of northern China
Source: PeerJ. 2018 Oct 9;6:e5741. doi: 10.7717/peerj.5741 (PMC6183509; doi:10.7717/peerj.5741)
Supplement: Figure S14 — The color of the branch represents its corresponding phylum, and each color represents a phylum. The size of the circle is proportional to the abundance of the taxonomic groups. The top 40 taxonomic groups in abundance are represented by solid circles. [file peerj-06-5741-s018.pdf]

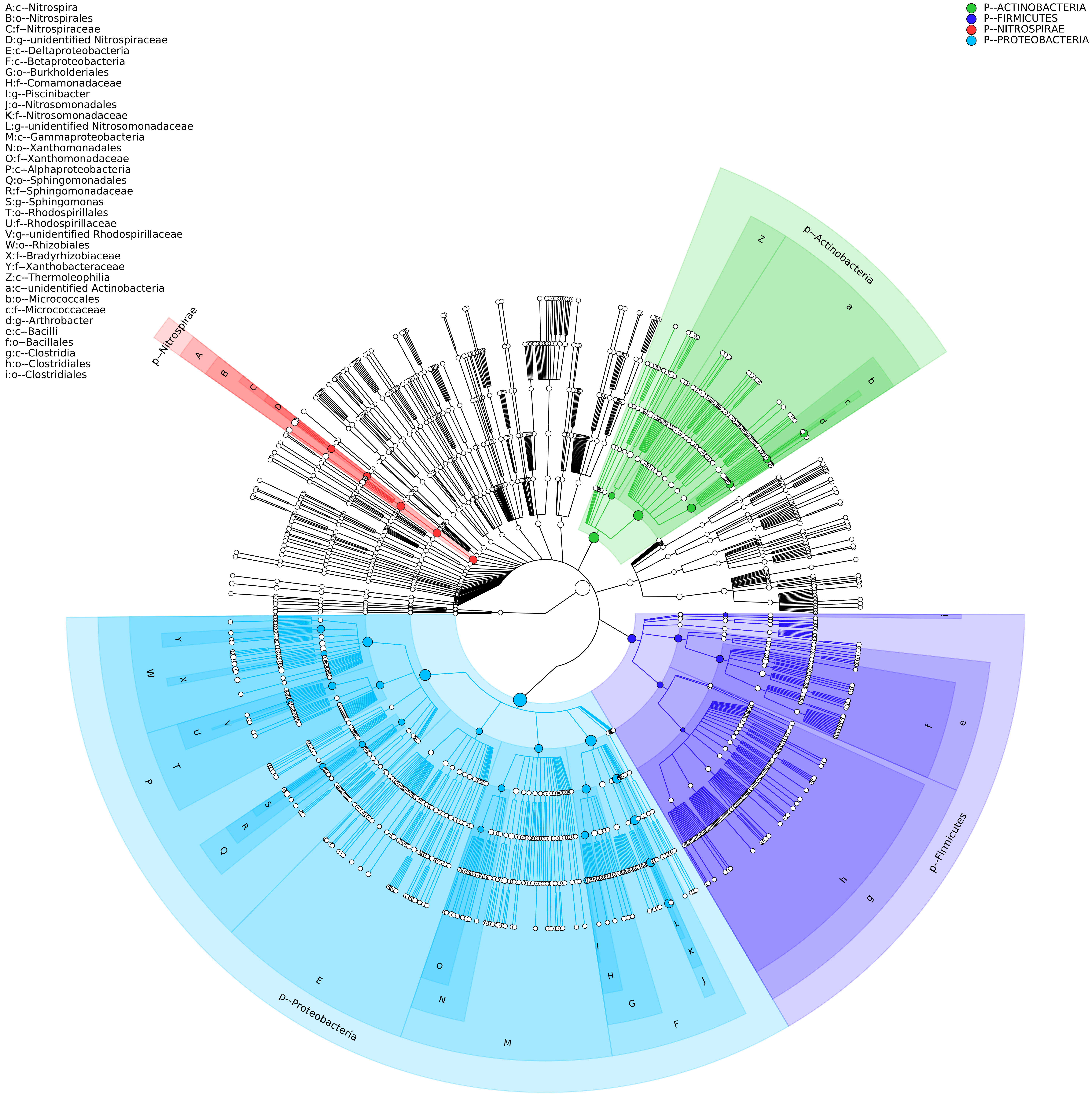
A:c--Nitrospira
B:o--Nitrospirales
C:f--Nitrospiraceae
D:g--unidentified Nitrospiraceae
E:c--Deltaproteobacteria
F:c--Betaproteobacteria
G:o--Burkholderiales
H:f--Comamonadaceae
I:g--Piscinibacter
J:o--Nitrosomonadales
K:f--Nitrosomonadaceae
L:g--unidentified Nitrosomonadaceae
M:c--Gammaproteobacteria
N:o--Xanthomonadales
O:f--Xanthomonadaceae
P:c--Alphaproteobacteria
Q:o--Sphingomonadales
R:f--Sphingomonadaceae
S:g--Sphingomonas
T:o--Rhodospirillales
U:f--Rhodospirillaceae
V:g--unidentified Rhodospirillaceae
W:o--Rhizobiales
X:f--Bradyrhizobiaceae
Y:f--Xanthobacteraceae
Z:c--Thermoleophilia
a:c--unidentified Actinobacteria
b:o--Micrococcales
c:f--Micrococcaceae
d:g--Arthrobacter
e:c--Bacilli
f:o--Bacillales
g:c--Clostridia
h:o--Clostridiales
i:o--Clostridiales
P--ACTINOBACTERIA
P--FIRMICUTES
P--NITROSPIRAE
P--PROTEOBACTERIA
p--Nitrospirae
p--Actinobacteria
p--Firmicutes
p--Proteobacteria
