# Supplementary material for: Effects of wine-cap Stropharia cultivation on soil nutrients and bacterial communities in forestlands of northern China
Source: PeerJ. 2018 Oct 9;6:e5741. doi: 10.7717/peerj.5741 (PMC6183509; doi:10.7717/peerj.5741)
Supplement: Figure S15 — The color of the branch represents its corresponding phylum, and each color represents a phylum. The size of the circle is proportional to the abundance of the taxonomic groups. The top 40 taxonomic groups in abundance are represented by solid circles. [file peerj-06-5741-s019.pdf]

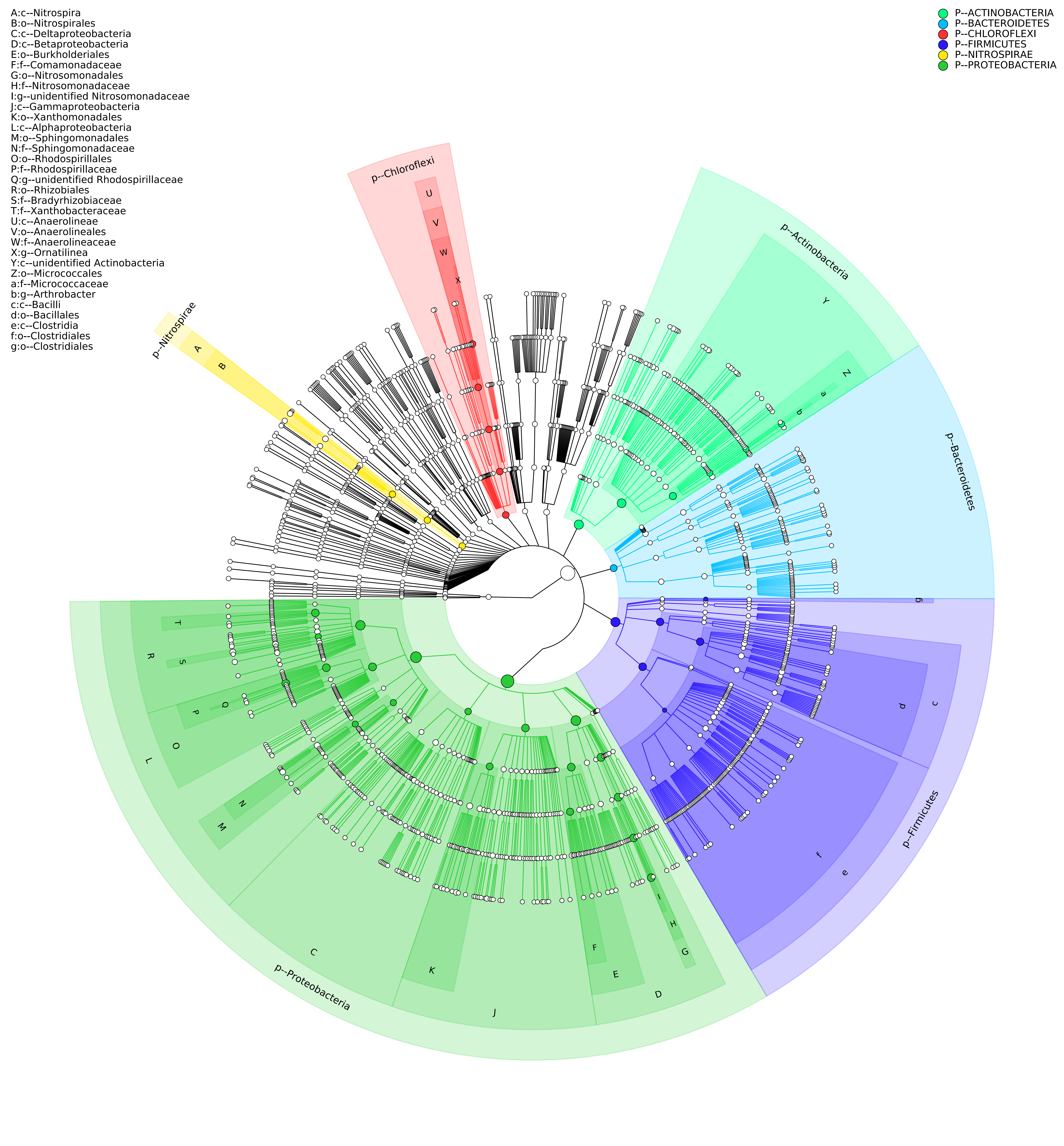
A:c--Nitrospira
B:o--Nitrospirales
C:c--Deltaproteobacteria
D:c--Betaproteobacteria
E:o--Burkholderiales
F:f--Comamonadaceae
G:o--Nitrosomonadales
H:f--Nitrosomonadaceae
I:g--unidentified Nitrosomonadaceae
J:c--Gammaproteobacteria
K:o--Xanthomonadales
L:c--Alphaproteobacteria
M:o--Sphingomonadales
N:f--Sphingomonadaceae
O:o--Rhodospirillales
P:f--Rhodospirillaceae
Q:g--unidentified Rhodospirillaceae
R:o--Rhizobiales
S:f--Bradyrhizobiaceae
T:f--Xanthobacteraceae
U:c--Anaerolineae
V:o--Anaerolineales
W:f--Anaerolineaceae
X:g--Ornatilinea
Y:c--unidentified Actinobacteria
Z:o--Micrococcales
a:f--Micrococcaceae
b:g--Arthrobacter
c:c--Bacilli
d:o--Bacillales
e:c--Clostridia
f:o--Clostridiales
g:o--Clostridiales
P--ACTINOBACTERIA
P--BACTEROIDETES
P--CHLOROFLEXI
P--FIRMICUTES
P--NITROSPIRAE
P--PROTEOBACTERIA
p--Nitrospirae
p--Chloroflexi
p--Actinobacteria
p--Bacteroidetes
p--Firmicutes
p--Proteobacteria
