# Supplementary material for: Effects of wine-cap Stropharia cultivation on soil nutrients and bacterial communities in forestlands of northern China
Source: PeerJ. 2018 Oct 9;6:e5741. doi: 10.7717/peerj.5741 (PMC6183509; doi:10.7717/peerj.5741)
Supplement: Figure S16 — The color of the branch represents its corresponding phylum, and each color represents a phylum. The size of the circle is proportional to the abundance of the taxonomic groups. The top 40 taxonomic groups in abundance are represented by solid circles. [file peerj-06-5741-s020.pdf]

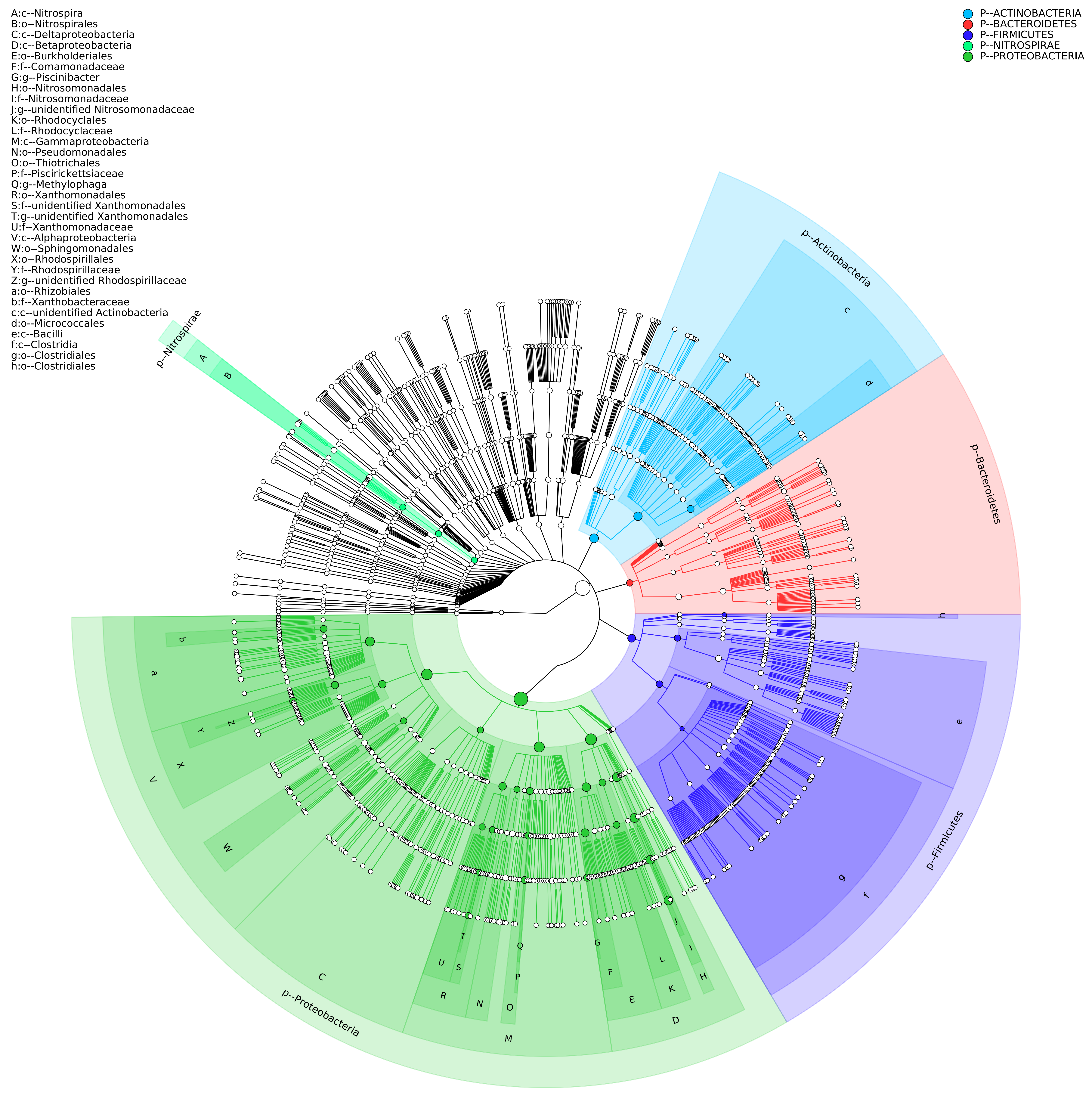
A:c--Nitrospira
B:o--Nitrospirales
C:c--Deltaproteobacteria
D:c--Betaproteobacteria
E:o--Burkholderiales
F:f--Comamonadaceae
G:g--Piscinibacter
H:o--Nitrosomonadales
I:f--Nitrosomonadaceae
J:g--unidentified Nitrosomonadaceae
K:o--Rhodocyclales
L:f--Rhodocyclaceae
M:c--Gammaproteobacteria
N:o--Pseudomonadales
O:o--Thiotrichales
P:f--Piscirickettsiaceae
Q:g--Methylophaga
R:o--Xanthomonadales
S:f--unidentified Xanthomonadales
T:g--unidentified Xanthomonadales
U:f--Xanthomonadaceae
V:c--Alphaproteobacteria
W:o--Sphingomonadales
X:o--Rhodospirillales
Y:f--Rhodospirillaceae
Z:g--unidentified Rhodospirillaceae
a:o--Rhizobiales
b:f--Xanthobacteraceae
c:c--unidentified Actinobacteria
d:o--Micrococcales
e:c--Bacilli
f:c--Clostridia
g:o--Clostridiales
h:o--Clostridiales
P--ACTINOBACTERIA
P--BACTEROIDETES
P--FIRMICUTES
P--NITROSPIRAE
P--PROTEOBACTERIA
p--Nitrospirae
p--Actinobacteria
p--Bacteroidetes
p--Firmicutes
p--Proteobacteria
