# Supplementary material for: Effects of wine-cap Stropharia cultivation on soil nutrients and bacterial communities in forestlands of northern China
Source: PeerJ. 2018 Oct 9;6:e5741. doi: 10.7717/peerj.5741 (PMC6183509; doi:10.7717/peerj.5741)
Supplement: Figure S17 — The color of the branch represents its corresponding phylum, and each color represents a phylum. The size of the circle is proportional to the abundance of the taxonomic groups. The top 40 taxonomic groups in abundance are represented by solid circles. [file peerj-06-5741-s021.pdf]

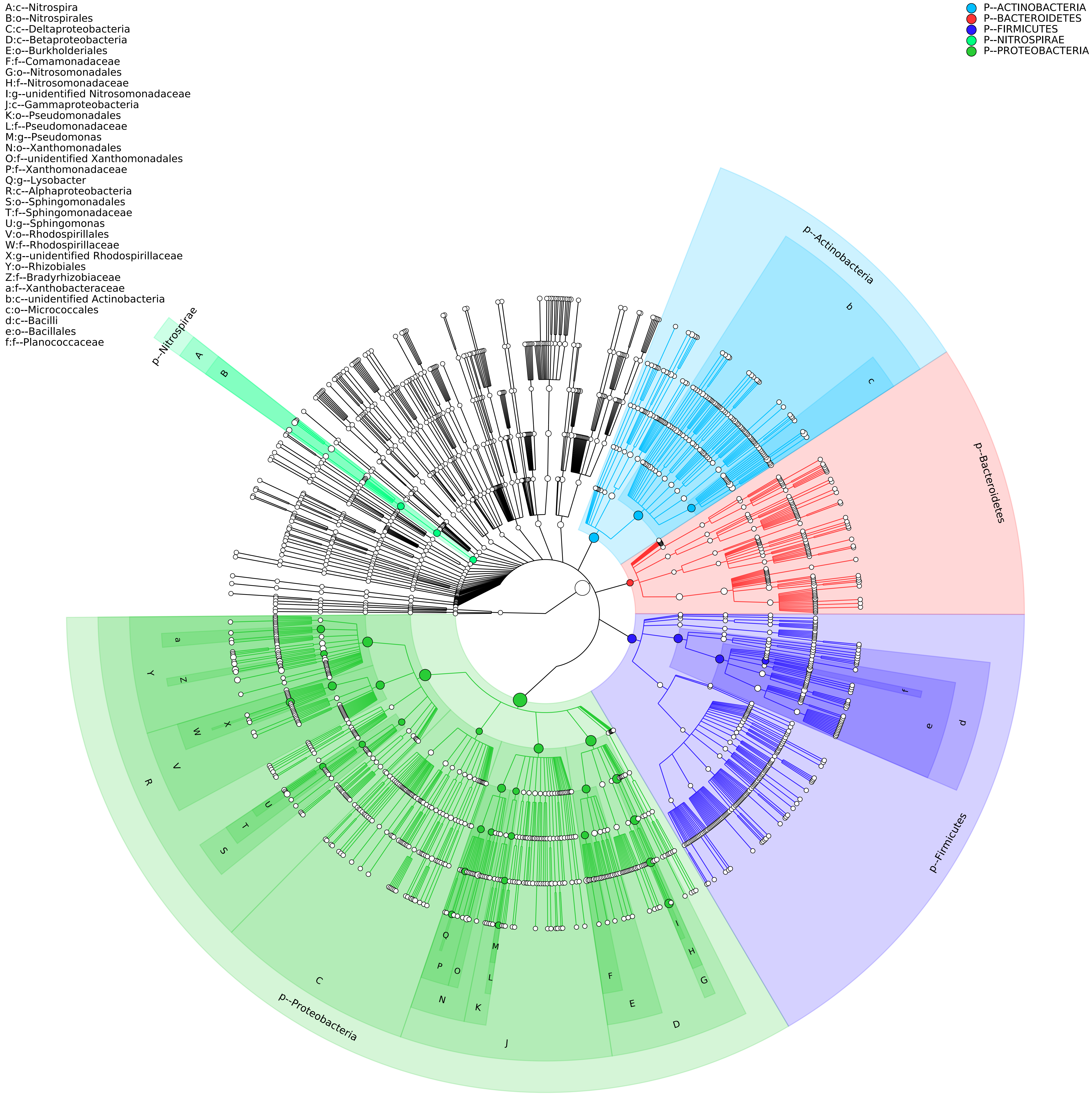
A:c--Nitrospira
B:o--Nitrospirales
C:c--Deltaproteobacteria
D:c--Betaproteobacteria
E:o--Burkholderiales
F:f--Comamonadaceae
G:o--Nitrosomonadales
H:f--Nitrosomonadaceae
I:g--unidentified Nitrosomonadaceae
J:c--Gammaproteobacteria
K:o--Pseudomonadales
L:f--Pseudomonadaceae
M:g--Pseudomonas
N:o--Xanthomonadales
O:f--unidentified Xanthomonadales
P:f--Xanthomonadaceae
Q:g--Lysobacter
R:c--Alphaproteobacteria
S:o--Sphingomonadales
T:f--Sphingomonadaceae
U:g--Sphingomonas
V:o--Rhodospirillales
W:f--Rhodospirillaceae
X:g--unidentified Rhodospirillaceae
Y:o--Rhizobiales
Z:f--Bradyrhizobiaceae
a:f--Xanthobacteraceae
b:c--unidentified Actinobacteria
c:o--Micrococcales
d:c--Bacilli
e:o--Bacillales
f:f--Planococcaceae
P--ACTINOBACTERIA
P--BACTEROIDETES
P--FIRMICUTES
P--NITROSPIRAE
P--PROTEOBACTERIA
p--Nitrospirae
p--Actinobacteria
p--Bacteroidetes
p--Firmicutes
p--Proteobacteria
